# Nuclear Receptor Pathways Mediating the Development of Boar Taint

**DOI:** 10.3390/metabo12090785

**Published:** 2022-08-25

**Authors:** Christine Bone, E. James Squires

**Affiliations:** Department of Animal Biosciences, University of Guelph, Guelph, ON N1G 2W1, Canada

**Keywords:** boar taint, metabolism, nuclear receptor, signaling pathways, cytochrome P450, PXR, CAR, FXR

## Abstract

The nuclear receptors PXR, CAR, and FXR are activated by various ligands and function as transcription factors to control the expression of genes that regulate the synthesis and metabolism of androstenone and skatole. These compounds are produced in entire male pigs and accumulate in the fat to cause the development of a meat quality issue known as boar taint. The extent of this accumulation is influenced by the synthesis and hepatic clearance of androstenone and skatole. For this reason, PXR, CAR, and FXR-mediated signaling pathways have garnered interest as potential targets for specialized treatments designed to reduce the development of boar taint. Recent research has also identified several metabolites produced by gut microbes that act as ligands for these nuclear receptors (e.g., tryptophan metabolites, short-chain fatty acids, bile acids); however, the connection between the gut microbiome and boar taint development is not clear. In this review, we describe the nuclear receptor signaling pathways that regulate the synthesis and metabolism of boar taint compounds and outline the genes involved. We also discuss several microbial-derived metabolites and dietary additives that are known or suspected nuclear receptor ligands and suggest how these compounds could be used to develop novel treatments for boar taint.

## 1. Introduction

Nuclear receptors are a family of transcription factors that regulate the expression of genes controlling numerous physiological processes [1,2]. All nuclear receptors share a common structure, which includes a DNA binding domain (DBD) and a C-terminal ligand-binding domain (LBD) [2]. The LBD contains a binding pocket that recognizes specific endogenous and exogenous ligands that activate the nuclear receptor upon binding [3]. The DBD contains zinc fingers, which recognize and bind to specific DNA sequences called response elements [4]. Nuclear receptors also contain a ligand-dependent activation function (AF-2) within the LBD and an N-terminal ligand-independent activation function (AF-1), which recruit coactivating proteins to synergistically regulate gene transcription [3,5,6,7,8,9].

The nuclear receptors pregnane X receptor (PXR), constitutive androstane receptor (CAR), and farnesoid X receptor (FXR) are class II nuclear receptors that form heterodimers with the retinoid X receptor (RXR) and bind to response elements primarily organized as direct repeats [1,10].

Initial studies characterized PXR as a ligand-dependent transcription factor for several genes belonging to the cytochrome P4503A (CYP3A) subfamily, activated by naturally occurring pregnane steroids such as pregnenolone and progesterone [11]. PXR is now known to regulate the expression of nearly 500 genes in human hepatocytes alone, and numerous ligands, including drugs, natural products, and endogenous ligands, such as secondary bile acids or metabolites produced by gut microbes, have been identified [12,13,14,15]. 

CAR was first described as a constitutive receptor and was found to be inactivated by 3α-hydroxy, 5α-reduced androstane steroids [16]. Several activators of CAR have since been identified, including phenobarbital, steroids such as 5β-pregnane-3,20-dione, several drugs, and natural products; however, they do not necessarily interact directly with the receptor and instead can regulate CAR activity by promoting nuclear translocation [12,17,18,19,20,21,22]. PXR and CAR are highly expressed in the liver and intestinal tract and are key regulators of xenobiotic metabolism [23,24]. 

The activation of FXR was first demonstrated in response to farnesol derivatives, and the receptor was named accordingly [25]. However, bile acids such as chenodeoxycholic acid (CDCA), cholic acid (CA), deoxycholic acid (DCA), lithocholic acid (LCA), and their conjugates were later identified as natural ligands for FXR under physiological conditions, with CDCA being the most effective agonist [10,26,27,28,29]. FXR is highly expressed in the liver, kidney, and intestinal villi and controls a wide spectrum of metabolic pathways; most notably, FXR mediates the autoregulatory effects of bile acids on bile acid homeostasis [25,30]. FXR can also function as a transcription factor for *PXR* and can modulate the production of bile acid metabolites that activate or repress PXR and CAR signaling pathways [31,32,33]. 

Porcine orthologues of PXR, CAR, and FXR share a high degree of sequence homology with human receptors and respond to similar ligands [34,35,36]. These nuclear receptors, as well as several splice variants, have been isolated from the liver and testis, where they have been shown to modulate the synthesis and metabolism of compounds produced in entire male pigs that are responsible for a meat quality issue known as boar taint [37,38]. Boar taint describes an off-odour or off-flavour that results from the accumulation of 5α-androst-16-ene-3-one (androstenone) and 3-methylindole (skatole) in the fat, which has traditionally been prevented by surgically castrating male pigs shortly after birth. However, surgical castration has come under increasing public scrutiny due to animal welfare concerns, and the negative environmental impact associated with raising surgical castrates, which consume more feed and grow less efficiently than entire male pigs [39]. For this reason, PXR, CAR, and FXR are potentially attractive targets for treatments designed to prevent boar taint without castration.

## 2. Nuclear Receptor Signaling and Boar Taint

### 2.1. Nuclear Receptor-Mediated Regulation of Testicular Steroid Synthesis

The testis of the boar is a dynamic organ that is the primary site of synthesis for various steroid hormones, including the 16-androstene steroid androstenone. Androstenone is a sex pheromone that is produced by the boar at the onset of puberty, which can regulate female reproductive behaviour. Like all steroids, androstenone is derived from the stepwise conversion of cholesterol. A key step in this pathway involves the conversion of pregnenolone to the 16-androstene steroid 5,16-androstadien-3β-ol (androstadienol), a precursor of androstenone [40]. This is catalyzed by the andien-β synthase activity of cytochrome P45017A1 (CYP17A1) and is regulated by several accessory proteins such as cytochrome b5A (CYB5A) and cytochrome b5 reductase (CYB5R3) [41,42]. 

**Figure 1:**
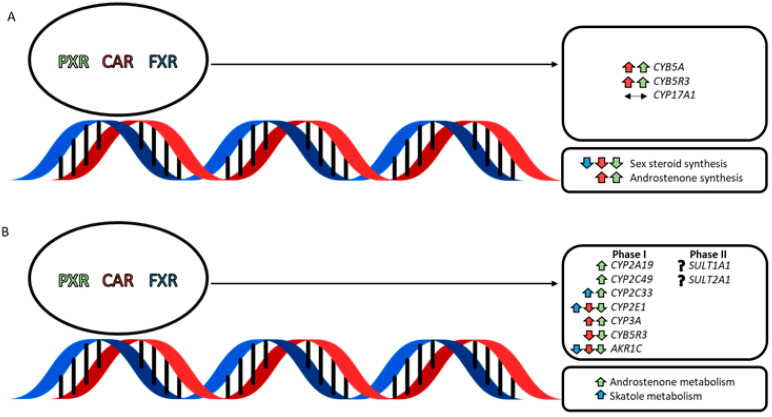
The effects of nuclear receptor activation reported by Gray and Squires [37,38] in porcine (**A**) Leydig cells [37] and (**B**) hepatocytes [38]. PXR, CAR, and FXR, shown in green, red, and blue, respectively, increase (↑) or decrease (↓) the expression of genes in the testis that regulate steroidogenesis and Phase I and II metabolism in the liver. Symbols in black represent no effect (↔) or no characterized effect (?) of PXR, CAR, or FXR on the genes of interest. The overall effect on the synthesis or metabolism of androstenone and skatole is shown.

Nuclear receptor activation in porcine Leydig cells (Figure 1A) has been shown to alter the synthesis of the 16-androstene steroids, which is presumably mediated by nuclear receptor-induced changes in the expression of *CYB5A* and *CYB5R3* [37]. Activation of PXR and CAR increased *CYB5A* and *CYB5R3* expression and decreased the production of sex steroids while increasing 16-androstene steroid synthesis. FXR activation also decreased the production of sex steroids but did not affect the synthesis of 16-androstene steroids [37]. The 17,20-lyase reaction that is required for the conversion of pregnenolone to sex steroids is also catalyzed by CYP17A1 and is prioritized over the andien-β synthase reaction when levels of CYB5A are limiting [43]. This suggests that the activation of PXR and CAR promotes the synthesis of 16-androstene steroids by upregulating *CYB5A* and *CYB5R3* to favour the andien-β synthase activity of CYP17A1.

### 2.2. Nuclear Receptor-Mediated Regulation of Boar Taint Metabolism

The liver is the primary site for the Phase I and Phase II metabolic reactions that promote the hepatic clearance of androstenone and skatole (Figure 1B). During Phase I metabolism, oxidation, reduction, or hydrolysis reactions result in an addition of a hydroxyl group to the compound, which can serve as a site for conjugation during Phase II metabolic reactions. Phase I oxidation reactions are modulated by the cytochrome P450 (CYP450) enzyme superfamily and the accessory proteins NADPH-cytochrome P450 oxidoreductase (POR) or CYB5A [44,45]. The total expression of hepatic CYP450s is similar between humans and pigs; however, differences in the relative quantity of CYP450 subfamilies have been reported [46]. In particular, the expression of the CYP2A, CYP2D, and CYP2E subfamilies are more abundant in pigs, while CYP1A2 and the CYP3A and CYP2C subfamilies are more abundant in humans [46].

#### 2.2.1. Nuclear Receptor-Mediated Effects on Skatole Metabolism

The porcine CYP450 isoforms CYP1A1, CYP2A19, CYP2C33v4, CYP2C49, CYP2E1, and CYP3A regulate the Phase I metabolism of skatole [45,47,48,49]. The Phase I metabolites of skatole with the most notable implications for the development of boar taint include 3-hydroxy-3-methyloxindole (HMOI), indole-3-carbinol (I3C), and 6-hydroxy-3-methylindole (6-OH-3MI), which is sulfoconjugated by the sulfotransferase enzyme SULT1A1 during Phase II metabolism to form 6-OH-3MI sulfate [50,51]. However, four additional metabolites have been identified and include 3-methyloxindole (3MOI), 5-hydroxy-3-methylindole (5-OH-3MI), 2-aminoacetophenone (2AAP), and 3-hydroxy-3-methylindolenine (3-OH-MI), which is the most abundant skatole metabolite produced during Phase I metabolism [52]. Higher plasma and urine concentrations of HMOI and urine concentrations of I3C have been noted in animals with high fat concentrations of skatole, while animals with low concentrations of skatole in the fat tend to produce greater concentrations of the metabolite 6-OH-3MI sulfate [51,53]. Therefore, HMOI, I3C, and 6-OH-3MI are important biomarkers for boar taint from skatole. 

Schelstraete et al. [46] reported that the protein expression of CYP2A19 and CYP2E1 in porcine hepatocytes accounted for nearly half of the total CYP450 protein quantified (31% and 13%, respectively). Achour et al. [54] observed similar relative protein expression profiles indicating that CYP2A19 and CYP2E1 are highly expressed in the liver of pigs. Both CYP2A19 and CYP2E1 produce large quantities of the key Phase I metabolite 6-OH-3MI relative to the other CYP450s regulating skatole metabolism and are downregulated in animals with high levels of androstenone and skatole the fat [45,48,51,55]. Androstenone has been shown to have an inhibitory effect on the protein expression and activity of CYP2E1 and CYP2A6, the human orthologue of CYP2A19 [48,56,57]. Therefore, the hepatic expression of *CYP2A19* and *CYP2E1* has important implications for the development of boar taint. CYP2C49 has also been identified as an important regulator of skatole metabolism as it produces significant quantities of 6-OH-3MI; however, *CYP2C49* is upregulated in animals with high fat androstenone concentrations along with *CYP2C33* [45,55].

Activation of PXR, but not CAR, has been found to upregulate the expression of *CYP2A19* in porcine hepatocytes [38], despite *CYP2A6* being a gene target for both nuclear receptors in humans [46,58]. PXR activation also increased the expression of *CYP2C49* and *CYP2C33*, while decreased expression of *CYP2E1* and *CYB5R3* resulted from the activation of both PXR and CAR. Puccinelli et al. [58] reported similar effects on gene expression following phenobarbital administration in vivo. Phenobarbital, a known activator of PXR and CAR, increased the expression and activity of CYP2C33, CYP2C49, and CYP3A in the liver of pigs. These changes in gene expression were associated with an increase production of I3C by activation of PXR and HMOI by activation of CAR in porcine hepatocyte culture [38]. The production of 6-OH-3MI was not altered despite the upregulation of *CYP2C49* and *CYP2A19* by PXR; however, the activity of these enzymes may have been reduced by the downregulation of *CYB5R3*, which works alongside CYB5A to increase the production of 6-OH-3MI by CYP2C49, CY2A19, and CYP2E1 [45]. In contrast, FXR activation in porcine hepatocytes upregulated the expression of *CYP2E1* without altering *CYB5R3* expression and significantly increased the production of 6-OH-3MI [38].

#### 2.2.2. Nuclear Receptor-Mediated Effects on Androstenone Metabolism

Phase I metabolites of androstenone include 3α-androstenol and 3β-androstenol, which are produced from the reduction in androstenone by aldo-keto reductase (AKR1C) or 3β-hydroxysteroid dehydrogenase (HSD3B), respectively [40]. These 16-androstene steroids are substrates for the sulfotransferase enzyme SULT2A1, which promotes Phase II sulfoconjugation reactions [59]. The metabolism of androstenone also occurs in the testis, and a negative correlation between testicular SULT2A1 activity and fat androstenone concentrations has been reported [60]. This suggests that the sulfoconjugation of androstenone and its metabolites during Phase II metabolism is essential for reducing the development of boar taint. However, we have recently shown that androstenone sulfate can be transported into the adipose tissue and deconjugated by the enzyme steroid sulfatase (STS) [61]. The deconjugation of androstenone sulfate was positively correlated with fat androstenone concentrations in early but not late-maturing boars, which suggests that the sulfoconjugation of the 16-androstene steroids may not reduce boar taint development in all animals. Thus, androstenone metabolism in the testis may primarily function to convert excess concentrations of androstenone to an inactive reservoir, which can be deconjugated during low periods of steroid synthesis to return free androstenone. Such is the case for sulfoconjugated estrogens in the boar [62]. In contrast, androstenone metabolism in the liver may instead favour hepatic clearance and excretion.

The activation of PXR has been found to significantly increase androstenone metabolism by porcine hepatocytes, but the mechanism behind this is not well understood. Gray and Squires [38] reported a significant decrease in the initial percentage of androstenone following treatment with rifampicin, a potent agonist of PXR in pigs and humans. The activation of PXR also decreased the expression of *AKR1C* but did not alter the production of the Phase I metabolite 3α-androstenol; however, 3β-androstenol synthesis was decreased. Interestingly, the expression of *AKR1C* was also decreased following CAR and FXR activation, but no effect on the metabolism of androstenone was observed. These results suggest that PXR activation may increase the metabolism of androstenone by promoting the synthesis of Phase II conjugated metabolites. 

In humans, the activation of PXR and CAR results in the upregulation of several genes that control Phase II metabolic reactions, including *SULT2A1*, and FXR activation induces the expression of *SULT1A1* [33,63]. However, the effect of nuclear receptor activation on the Phase II metabolism of androstenone and skatole has not been reported. Therefore, future research is needed to evaluate the effect of PXR, CAR, and FXR activation on the expression of genes regulating Phase II metabolic reactions and the production of conjugated metabolites of androstenone and skatole. 

### 2.3. Coregulatory Proteins and Nuclear Receptor Crosstalk

The effect of nuclear receptor activation on the hepatic metabolism of boar taint compounds may also be influenced by the availability of different nuclear receptor coactivators (NCOAs) and nuclear receptor corepressors (NCORs) that regulate the transcriptional activity of PXR, CAR, and FXR. 

NCOA1, NCOA2, and NCOA3 are well-characterized members of the p160 steroid receptor coactivator (SRC) protein family that are recruited to the enhancer region of target genes by ligand-activated nuclear receptors to promote gene transcription [5,64]. NCOAs share a common structure: an N-terminal basic helix–loop–helix-PER-ARNT-SIM (bHLH/PAS) domain, a serine/threonine rich domain, an “NR” box containing LXXLL motifs, which are recognized by the AF-2 region within the nuclear receptor LBD, and two C-terminal transactivation domains (AD1/AD2) [65,66,67]. Within each domain, there are sites for various post-translational modifications, including acetylation, phosphorylation, methylation, ubiquitination, and sumoylation [68,69,70]. NCOAs form multi-subunit coactivator complexes by recruiting secondary coactivator proteins (e.g., protein arginine methyltransferase 1 (PRMT1), coactivator-associated arginine methyltransferase 1 (CARM1), and CBP/p300), which remodel chromatin and modify histones to promote gene transcription [64,65,71]. 

In contrast, nuclear receptors recruit NCORs to the repressor region of target genes in the absence of ligand binding to inhibit gene transcription. The NCORs include NCOR1 and NCOR2, which is better known as silencing mediator for retinoid and thyroid hormone receptor (SMRT) [65]. NCOR1 contains a deacetylase activating domain (DAD), three N-terminal repression domains (RD), and three C-terminal “CoRNR” box motifs (LXX I/H I XXX I/L), which serve as sites for nuclear receptor interaction. SMRT is similar in structure but contains an additional RD and one less nuclear receptor interaction site [72,73,74]. NCORs exert their inhibitory effect on gene transcription through interactions with histone deacetylase 3 (HDAC3) and transducin β-like 1 X-linked/transducin β-like 1 X-liked receptor 1 (TBL1X/TBL1XR1), which bind within the DAD and RDs, respectively, as well as the recruitment of additional corepressor complexes [70,74]. 

Nuclear receptor crosstalk can also regulate the transcriptional activity of PXR, CAR, and FXR and this may be important to consider for future studies investigating the effect of nuclear receptor activation on the development of boar taint. Recent studies have demonstrated that activation of FXR in the testis can induce the transcription of dosage-sensitive sex reversal, adrenal hypoplasia critical region, on chromosome X, gene-1 (DAX-1) or small heterodimer partner (SHP) to repress steroidogenesis in pubertal mice [31,75]. In the liver, SHP represses the transcriptional activity of PXR, CAR, and FXR by inhibiting interactions with coactivator proteins; however, PXR crosstalk can regulate SHP expression [71]. PXR inhibits the recruitment of peroxisome proliferator-activated receptor-gamma coactivator (PGC)-1α by hepatocyte nuclear factor 4 alpha (HNF4α) to repress *SHP* transcription, which restores interactions between nuclear receptors and coactivator proteins to promote the transcription of various target genes [71,76,77]. Competition between nuclear receptors or transcription factors for common coregulatory proteins is another source of nuclear receptor crosstalk, and this occurs between PXR and CAR, which compete for the common coactivator NCOA1 [78]. Specific ligands can also differentially regulate nuclear receptor signaling pathways and promote nuclear receptor crosstalk. For example, PXR and FXR have several common agonists (e.g., bile acids such as CA, 12-ketolithocholic acid, and some bile acid derivatives), which have an inhibitory effect on CAR activity [79]. Additionally, nuclear receptors can interact with different coactivator proteins in a ligand-dependent manner [80]. Therefore, further work is required to characterize the impact of coregulatory proteins and crosstalk events on the development of boar taint in pigs. 

## 3. The Gut–Liver Axis

The gut microbiome is a complex organ that is typically comprised of a couple hundred bacterial species expressing nearly 2 million different genes, which promote the biotransformation of xenobiotics and endogenous compounds and regulate the production of microbial metabolites in response to dietary, genetic, and environmental factors [81,82]. Microbiota-derived compounds function as signaling molecules between different bacterial species to synchronize bacterial behaviours by altering the microbial population or the gene expression within the gut microbiome, which is known as quorum sensing [83]. Gut-derived compounds also modulate metabolic pathways in the liver and intestines and act as ligands for nuclear receptors and other xenobiotic sensing transcription factors [82,84]. In response, the liver produces bile to provide feedback to the gut microbiota and regulate further metabolite production [85]. This bidirectional communication between the liver and the gut is referred to as the gut–liver axis and represents an important link between the gut microbiome and nuclear receptor signaling pathways. Despite this, research examining the relationship between the gut–liver axis and the development of boar taint is surprisingly limited. 

Several studies have suggested that the metabolism of boar taint compounds may occur in extra-hepatic tissues. PXR, CAR, and FXR are constitutively expressed in the porcine intestinal tract along with CYP1A1, CYP3A29, CYP3A22, and CYP3A46 [34,35,86]. Moreover, dietary supplementation with phenobarbital was reported to induce additional CYP450s in porcine enterocytes such as CYP2C49 and CYP2C33, and it has been suggested that ligands for other nuclear receptors or transcription factors may induce different skatole metabolizing CYP450s such as CYP2E1 [58,86]. This suggests that the activation of nuclear receptors by microbial-derived compounds may regulate the metabolism of boar taint compounds in the intestinal tract and liver; however, the combined effect of extra-hepatic and hepatic metabolism on the development of boar taint is not well established. 

### 3.1. Gut-Derived Tryptophan Metabolites

Indole-3-propionic acid (IPA) is an indole derivative synthesized from the reductive metabolism of tryptophan in the gut [87]. In this reductive pathway, tryptophan is converted to indole-3-lactic acid (ILA), indole-3-acrylic acid (IA), and IPA by several species of *Clostridium* and *Peptostreptococcus* expressing the phenyllactate dehydratase gene cluster (*fldAIBC*) [87,88,89]. IPA is an endogenous ligand and activator of PXR, which primarily regulates PXR signaling pathways within the gut to improve gastrointestinal barrier function [14]. IPA is also a ligand for the aryl hydrocarbon receptor (AhR), a ligand-dependent transcription factor that shares 88% of its known activators with PXR [90]. AhR is also expressed in the liver and intestinal tract of pigs, and crosstalk between AhR and PXR was previously found to mediate the cytostatic properties of IPA in breast cancer cells [86,91]. However, PXR signaling is also regulated by negative crosstalk with AhR, which may explain why IPA induces AhR, but not PXR, target genes in the liver of mice [92]. 

AhR is activated by several other microbial-derived metabolites of tryptophan, including indole and indole-3-acetamide (IAD), which were recently identified as low- and medium- affinity ligands of human PXR, respectively [93]. Indole is produced from the hydrolysis of tryptophan by over 85 different bacterial species expressing tryptophanase [94], and the conversion of tryptophan to IAD is catalyzed by tryptophan-2-monooxygenase [95]. Illés et al. [93] reported that indole and IAD bind directly to the LBD of PXR and induce the PXR target genes, CYP3A4 and multidrug resistance 1 (MDR1), in human intestinal LS180 cells as well as CYP3A4 in primary human hepatocytes. Moreover, the intestinal anti-inflammatory properties of IPA via PXR are significantly enhanced in the presence of indole [14]. 

Skatole is also a microbial-derived metabolite of tryptophan that is produced in response to the transient accumulation of indole acetic acid (IAA) in the hindgut of pigs [96]. Numerous bacterial species modulate the conversion of tryptophan to IAA, and several tryptophan metabolites have been identified as precursors of IAA in mice [97]. However, the production of skatole from IAA is limited to four bacterial species in pigs that belong to the *Clostridium* and *Olsenella* genera [98,99]. Skatole is a low affinity ligand and partial agonist of human PXR, but a strong inverse agonist of PXR, CAR, and FXR in pigs [34,100]. Based on this, skatole may indirectly regulate boar taint development by suppressing nuclear receptor signaling pathways that promote the metabolism of boar taint compounds, in addition to accumulating in the fat directly. However, Gray and Squires reported contradictory effects of skatole in primary porcine Leydig cells [37] and hepatocytes [38]. Skatole decreased the expression of *CYP2B22*, the porcine orthologue of *CYP2B6*, and *CYB5R1* in Leydig cells and altered the ratio of 3α/3β-androstenol production by hepatocytes. However, skatole did not affect the total production of 16-androstene or sex steroids, nor the metabolism of androstenone in the testis and liver, respectively. Moreover, skatole did not alter the expression of several genes induced by activators of PXR, CAR, and FXR in both Leydig cells and hepatocytes, suggesting that crosstalk with other transcription factors may influence the suppressive effect of skatole on nuclear receptor signaling pathways. 

Skatole is a weak activator of AhR in humans and was found to decrease the mRNA expression of PXR in HepaRG cells along with several nuclear receptor target genes, including *CYP3A4*, *CYP2B6*, and *CYP2A6*, and to inhibit the induction of *CYP3A4* by rifampicin. However, the activation of AhR was proposed to de-regulate an unidentified factor mediating crosstalk between AhR, PXR, and basal CYP expression as skatole decreased the expression of CYP2E1, which is not a known target gene of PXR [90]. It is unclear if skatole is a ligand for AhR in pigs; however, the induction of CYP1A by a standard activator of AhR (β-napthoflavone) was demonstrated in primary porcine hepatocyte culture and was presumed to result from the activation of AhR [101,102]. Thus, future research should investigate the skatole-mediated activation of AhR in pigs, and potential crosstalk that is established with other nuclear receptor signaling pathways, to better understand the impact of skatole on the metabolism of boar taint compounds.

### 3.2. Short-Chain Fatty Acids

Acetate, propionate, and butyrate are the primary short-chain fatty acids (SCFAs) produced from the microbial fermentation of dietary fibre (e.g., pectin, hemicellulose, lignin, inulin, resistant starch) by anaerobic bacteria in the hindgut [103,104]. As extracellular signaling molecules, SCFAs target G-protein coupled receptors (GPR41, GPR43, GPR109a) to regulate protein kinase-dependent intracellular signaling pathways in the liver and the gut [105,106]. Moreover, propionate and butyrate inhibit histone deacetylases (HDACs) to regulate gene transcription [107]. This suggests that there are several opportunities for crosstalk between SCFAs and nuclear receptor signaling pathways. Interestingly, methoxyacetic acid and valproic acid, which are xenobiotics derived from the SCFAs acetate and valerate, respectively, have been found to enhance the activity of several steroid-activated nuclear receptors (e.g., estrogen receptor, progesterone receptor) via crosstalk involving mitogen-activated protein kinase signaling and inhibition of histone deacetylase [108]. Acetate, propionate, and butyrate induced histone acetylation and CYP1A1 in both Caco-2 and YAMC cells and enhanced the recruitment of AhR to the promoter [109]. Moreover, the effects of 1,4-dihydroxy-2-naphthoic acid (DHNA), a known activator of AhR, were enhanced in mice cotreated with butyrate and resulted in a 50-fold induction of CYP1A1 in the liver [109]. Butyrate was also reported to regulate CYP1A1 expression directly as a ligand and activator of AhR in human intestinal cells and was shown to induce PXR expression in Caco-2 cells [110,111]. This suggests that SCFAs such as butyrate may regulate nuclear receptor signaling pathways to control the metabolism of boar taint compounds.

### 3.3. Bile Acids

The primary bile acids CDCA and CA are cholesterol metabolites produced in the liver. Following synthesis, the primary bile acids are conjugated with glycine or taurine and incorporated into the bile. CDCA and CA are deconjugated by bile salt hydrolase enzymes, which are expressed by many bacterial species, including *Lactobacillus* [112,113,114], *Enterococcus* [115], *Bifidobacterium* [116,117], *Clostridium* [118], and *Bacteroides* [119]. Following deconjugation, approximately 95% of the bile acids released into the gut are re-absorbed and transported back to the liver via the hepatic portal vein bound to albumin or lipoproteins in what is known as the enterohepatic circulation [120,121,122]. Bile acids that escape re-absorption are metabolized in the colon by bacterial flora with 7α-dehydroxylation activity, resulting in the production of the secondary bile acids DCA and LCA from CA and CDCA, respectively [123,124]. 

As endogenous ligands and activators of FXR, bile acids induce the expression of several enzymes in the liver and gastrointestinal tract to autoregulate subsequent bile acid synthesis, transport, and metabolism and mitigate their potential cytotoxic effects. Upon activation, FXR increases the expression of SHP, which interacts with HNF4α and liver receptor homolog-1 (LRH-1) to inhibit CYP7A1 expression and bile acid synthesis [125]. Bile acids also work through FXR to directly increase the transcription of PXR, which functions as a target receptor for LCA and DCA [32,126]. The bile acid-mediated activation of FXR and PXR upregulates the expression of SULT2A1, UGT2B4, and CYP3A4 to promote bile acid metabolism/detoxification [127,128,129]. Some bile acids, bile acid conjugates, and bile acid metabolites also have inhibitory effects on CAR activity in humans and mice [130]. The metabolism of androstenone and skatole is dependent on many of the same hydroxylation and conjugation reactions that promote bile acid detoxification. Therefore, circulating levels of bile acids may indirectly affect the development of boar taint.

Like bile acids, androstenone is also thought to be recycled in the gut through the enterohepatic circulation. The inclusion of non-nutritive sorbent materials, most notably activated charcoal, in finishing diets was previously reported to significantly decrease fat androstenone concentrations [131]. While the mechanism behind this is unclear, it was proposed that dietary sorbent materials may disrupt the enterohepatic circulation of androstenone to promote excretion. This suggests that dietary sorbent materials may also indirectly alter nuclear receptor signaling pathways by reducing circulating levels of bile acids and bile acid derivatives. Therefore, future research aimed at characterizing the disruption of the enterohepatic circulation by dietary sorbent materials should also consider the potential effect of these binding agents on the activation or inhibition of PXR, CAR, and FXR, and the downstream consequences on the metabolism of androstenone and skatole.

### 3.4. Diet

The production of microbiota-derived compounds in the gut is highly dependent on the composition of the diet, and several dietary compounds have been investigated as a treatment strategy for boar taint. Most notably, raw potato starch, sugar beet pulp, chicory inulin, and other fermentable fibre sources can significantly reduce the synthesis of skatole in the hindgut; however, the exact mechanism behind this is not well understood. 

Claus et al. [132] attributed the effects of fermentable carbohydrates to the production of butyrate, which was shown to act in the gastrointestinal tract to inhibit apoptosis of colon crypt cells and reduce the production of cell debris that would otherwise provide a source of tryptophan for skatole synthesis. However, opposite effects on skatole synthesis and apoptosis have been reported following butyrate treatment via intracecal infusion [133]. Interestingly, butyrate can promote either growth stimulatory or apoptotic effects in human colorectal tumour cell lines in the absence and presence of glucose, respectively [134]. Therefore, this may explain the controversial effects of butyrate on skatole synthesis. 

Diets containing high levels of sugar beet pulp or chicory root effectively decrease fecal skatole concentrations and simultaneously increase the synthesis of IPA [133,135]. Although it has been suggested that fermentable carbohydrates may alter the microbial metabolism of tryptophan to favour the synthesis of IPA over skatole, IPA may alternatively act through nuclear receptor signaling pathways to promote skatole metabolism and clearance. Although the exact mechanism is unclear, the hepatic expression of CYP2E1 was increased in pigs fed sugar beet pulp [136]. Moreover, dietary supplementation with dried chicory root was reported to significantly increase the hepatic expression of CYP1A2 and CYP2A19 at the mRNA and protein level and CYP2E1 at the mRNA level relative to boars fed a standard control diet [137]. This suggests a possible link between IPA synthesis from the fermentation of dietary fibre, nuclear receptor activation, and boar taint metabolism. However, chicory root contains several sesquiterpene lactones, and some of these compounds (e.g., artemisinin) are established nuclear receptor agonists [138], which may alternatively explain these results. 

In addition to chicory root, several plant species and herbal medicines contain active compounds capable of selectively regulating nuclear receptor signaling pathways [139,140]. For example, oleanolic acid is a selective modulator of FXR found in many plant species and is used in Chinese herbal medicine for its hepatoprotective and anti-inflammatory effects [141]. Diallyl sulfide is an active ingredient found in garlic and an agonist of CAR, which has been reported to inhibit the activity of CYP2E1 in vivo and induce the expression of several CYP450s, including CYP1A, CYP2B, and CYP3A [20,141,142,143]. Moreover, hyperforin, from *Hypericum perforatum* or St. John’s Wort, is a high-affinity agonist of PXR [144], and the phytoestrogen coumestrol is a PXR antagonist [145]. Several compounds are also targets for multiple nuclear receptors, including ginkgolide A, a terpenoid found in *Ginkgo biloba*, which is an agonist of both PXR and CAR [146,147,148] and (Z)-guggulsterone, a plant sterol found in guggul plant (*Commiphora mukul*) resin, which is an agonist of PXR, antagonist of FXR, and inverse agonist of CAR [149,150]. Many natural products also contain fermentable carbohydrates that can increase the production of SCFAs, and several have been reported to modulate the composition of the gut microbiome (reviewed in [151]). Therefore, natural products may be a promising dietary treatment strategy for preventing the development of boar taint.

## 4. Recommendations for Future Research

Nuclear receptor signaling pathways have been extensively studied and well characterized in humans and mice, but studies investigating these effects in pigs are limited. Few studies have examined the impact of nuclear receptor transactivation on the synthesis and metabolism of boar taint compounds. The studies aimed at understanding these effects in pigs have predominantly been performed in vitro using primary cell culture systems, and only a limited number of agonists for PXR, CAR, and FXR have been investigated. These compounds have typically included rifampicin and 6-(4-Chlorophenyl)imidazo[2,1-b] [1,3] thiazole-5-carbaldehyde O-3,4-dichlorobenzyl) oxime (CITCO), which are pharmaceutical agents and the primary bile acid CDCA. Future research is needed to establish the effect of selective nuclear receptor activation on the synthesis and metabolism of androstenone and skatole in vivo to determine the efficacy of this method as a treatment for boar taint. However, the potential cytotoxic effects of dietary supplementation with rifampicin, CITCO, or CDCA have not been well characterized in a whole animal system. 

In a clinical trial, 10 mg/kg of rifampicin was provided as a treatment for patients with primary biliary cirrhosis daily over 14 months; however, 37.5% of the patients withdrew from the study after six months of treatment due to rifampicin-induced side effects [152]. CITCO (20 mg/kg) has been safely provided to hCAR-transgenic mice over six days as an adjuvant to enhance the tumour suppressing effects of the chemotherapy combination “CHOP”, which contains cyclophosphamide, doxorubicin, vincristine, and prednisone [153]. Moreover, a dietary inclusion level of 200 mg/kg for CDCA was previously found to increase body weight and average daily gain and improve intestinal health in piglets over 30 days [154]. However, it is not clear if these treatment durations would be sufficient for the prevention of boar taint or if these inclusion levels could be safely used in different species or older animals, as the hepatic expression of nuclear receptors and CYP450 enzymes increases significantly at the onset of puberty [155]. The use of these compounds may also create concerns regarding food safety due to the potential presence of drug residues in pork products from treated animals. Therefore, natural products may be a more suitable treatment for the prevention of boar taint, but in vitro studies are first needed to investigate the ability of these natural product compounds to selectively activate or inhibit porcine PXR, CAR, or FXR-mediated signaling pathways, which control the synthesis and metabolism of androstenone and skatole. This will allow for the identification of compounds that can maximize the metabolism of androstenone and skatole in the liver, without compromising the synthesis of sex steroids or increasing androstenone production in the testis. These studies should also consider the effect of coregulatory proteins and crosstalk events that may alter the response to treatment in a whole animal model in the presence of other endogenous ligands, which regulate the same or similar signaling pathways. 

Numerous factors and physiological systems differentially regulate the development of boar taint in individual animals. Consequently, the response to most existing treatments for boar taint varies significantly between individual animals. We have summarized these factors and their impact in a recent review [156]. This suggests that nuclear receptor transactivation may only regulate the synthesis and metabolism of boar taint compounds in a subset of animals, and this aspect should be carefully considered for future studies. The use of a defined whole animal model would allow for the identification of biomarkers that are common amongst animals that respond favourably to treatment. This could be accompanied with 16S rRNA sequencing to identify microbial communities in the gut that may act through the gut–liver axis to promote favourable treatment outcomes. The production of microbial metabolites in response to treatment should also be quantified using appropriate analytical methods including mass spectrometry or chromatography. Moreover, an interaction between the gut microbiome and the genome of the host should be considered due to the complex nature of boar taint. Transcriptome analysis using RNA sequencing has recently led to the identification of novel biomarkers and expression quantitative trait loci (eQTLs), which correspond to concentrations of androstenone and skatole in the fat [157,158,159]. These genetic markers complement the various candidate genes associated with high and low levels of boar taint that have previously been identified in the testis and liver (reviewed in [160]) and may also be useful for developing a gene profile associated with a favourable treatment outcome to reduce boar taint. 

## 5. Conclusions

Nuclear receptor signaling pathways can regulate various physiological processes, from bile acid homeostasis to detoxifying drugs and other exogenous compounds. There is also evidence to suggest that the nuclear receptors PXR, CAR, and FXR regulate the expression of genes controlling the synthesis and metabolism of compounds that are responsible for the development of boar taint. However, these effects need to be evaluated using whole animal studies. Several endogenous compounds such as tryptophan metabolites, SCFAs, or bile acids, which are produced or metabolized by microbes in the gut, have been suggested to alter nuclear receptor signaling pathways. Based on this, it may be possible to prevent the development of boar taint by providing dietary sources of fermentable carbohydrates and related compounds to promote the activation or inhibition of PXR, CAR, or FXR-mediated signaling pathways by altering the basal production of these endogenous compounds. Several natural products commonly found in plants have been identified as nuclear receptor ligands in humans and mice. These compounds could potentially be included as a dietary supplement to selectively activate or inhibit nuclear receptor-mediated signaling events for the prevention of boar taint.

## References

[1] Mangelsdorf D.J., Thummel C., Beato M., Herrlich P., Schutz G., Umesono K., Blumberg B., Kastner P., Mark M., Chambon P. (1995). The Nuclear Receptor Superfamily: The Second Decade. Cell.

[2] Sever R., Glass C.K. (2013). Signaling by Nuclear Receptors. Cold Spring Harb. Perspect. Biol..

[3] Bain D.L., Heneghan A.F., Connaghan-Jones K.D., Miura M.T. (2007). Nuclear Receptor Structure: Implications for Function. Annu. Rev. Physiol..

[4] Sugiura Y. (2001). Natural and Artificial Zinc Finger Proteins. RIKEN Rev..

[5] Xu J., Li Q. (2003). Review of the In Vivo Functions of the P160 Steroid Receptor Coactivator Family. Mol. Endocrinol..

[6] Kraus W.L., McInerney E.M., Katzenellenbogen B.S. (1995). Ligand-Dependent, Transcriptionally Productive Association of the Amino-and Carboxy-Terminal Regions of a Steroid Hormone Nuclear Receptor. Proc. Natl. Acad. Sci. USA..

[7] Tasset D., Tora L., Fromental C., Scheer E., Chambon P. (1990). Distinct Classes of Transcriptional Activating Domains Function by Different Mechanisms. Cell.

[8] Benecke A., Chambon P., Gronemeyer H. (2000). Synergy Between Estrogen Receptor an Activation Functions AF1 and AF2 Mediated by Transcription Intermediary Factor TIF2. EMBO Rep..

[9] Frigo D.E., Bondesson M., Williams C. (2021). Nuclear Receptors: From Molecular Mechanisms to Therapeutics. Essays Biochem..

[10] Wang Y.D., Chen W.D., Moore D.D., Wendong H. (2008). FXR: A Metabolic Regulator and Cell Protector. Cell Res..

[11] Kliewer S.A., Moore J.T., Wade L., Staudinger J., Watson M.A., Jones S.A., McKee D.D., Oliver B.B., Wilson T.M., Zetterström R.H. (1998). An Orphan Nuclear Receptor Activated by Pregnanes Defines a Novel Steroid Signaling Pathway. Cell.

[12] di Masi A., De Marnis E., Ascenzi P., Marino M. (2009). Nuclear Receptors CAR and PXR: Molecular, Functional and Biomedical Aspects. Mol. Asp. Med..

[13] Smutny T., Mani S., Pavek P. (2013). Post-Translational and Post-Transcriptional Modifications of Pregnane X Receptor (PXR) in Regulation of the Cytochrome P450 Superfamily. Curr. Drug Metab..

[14] Venkatesh M., Mukherjee S., Wang H., Li H., Sun K., Benechet A.P., Qiu Z., Maher L., Redinbo M.R., Phillips R.S. (2014). Symbiotic Bacterial Metabolites Regulate Gastrointestinal Barrier Function Via the Xenobiotic Sensor PXR and Toll-Like Receptor 4. Immunity.

[15] Motta S., Callea L., Tagliabue S.G., Bonati L. (2018). Exploring the PXR Ligand Binding Mechanism with Advanced Molecular Dynamics Methods. Sci. Rep..

[16] Forman B.M., Tzameli I., Choi H., Chen J., Simha D., Seol W., Evans R.M., Moore D.D. (1998). Androstane Metabolites Bind to And Deactivate the Nuclear Receptor CAR-β. Nature.

[17] Sueyoshi T., Kawamoto T., Zelko I., Honkakoski P., Negishi M. (1999). The Repressed Nuclear Receptor CAR Responds to Phenobarbital in Activating the Human CYP2B6 gene. J. Biol. Chem..

[18] Moore L.B., Parks D.J., Jones S.A., Bledsoe R.K., Consler T.G., Stimmel J.B., Goodwin B., Liddle C., Blanchard S.G., Wilson T.M. (2000). Orphan Nuclear Receptors Constitutive Androstane Receptor and Pregnane X Receptor Share Xenobiotic and Steroid Ligands. J. Biol. Chem..

[19] Tzamelia I., Pissios P., Schuetz E.G., Moore D.D. (2000). The Xenobiotic Compound 1,4-Bis[2-(3,5-Dichloropyridyloxy)]Benzene Is an Agonist Ligand for the Nuclear Receptor CAR. Mol. Cell. Biol..

[20] Fisher C.D., Augustine L.M., Maher J.M., Nelson D.M., Slitt A.L., Klaassen C.D., Lehman-Mckeeman L.D., Cherrington N.J. (2007). Induction of Drug-Metabolizing Enzymes by Garlic and Allyl Sulfide Compounds Via Activation of Constitutive Androstane Receptor and Nuclear Factor E2-Related Factor 2. Drug Metab. Dispos..

[21] Shizu R., Min J., Sobhany M., Pedersen L.C., Mutoh S., Negishi M. (2018). Interaction of the Phosphorylated DNA-Binding Domain in Nuclear Receptor CAR With Its Ligand-Binding Domain Regulates CAR Activation. J. Biol. Chem..

[22] Pham B., Arons A.B., Vincent J.G., Fernandez E.J., Shen T. (2019). Regulatory Mechanics of Constitutive Androstane Receptors: Basal and Ligand-Directed Actions. J. Chem. Inf. Model..

[23] Wei P., Zhang J., Egan-Hafley M., Liand S., Moore D. (2000). The Nuclear Receptor CAR Mediates Specific Xenobiotic Induction of Drug Metabolism. Nature.

[24] Chang T.K.H., Waxman D.J. (2006). Synthetic Drugs and Natural Products as Modulators of Constitutive Androstane Receptor (Car) And Pregnane X Receptor (PXR). Drug Metab. Rev..

[25] Forman B.M., Goode E., Chen J., Oro A.E., Bradley D.J., Perlman T., Noonan D.J., Burka L.T., McMoris T., Lamph W.W. (1995). Identification of a Nuclear Receptor That Is Activated by Farnesol Metabolites. Cell.

[26] Wang H., Chen J., Hollister K., Sowers L.C., Forman B.M. (1999). Endogenous Bile Acids Are Ligands for the Nuclear Receptor FXR/BAR. Mol. Cell.

[27] Makishima M., Okamoto A.Y., Repa J.J., Tu H., Learned R.M., Luk A., Hull M.V., Lustig K.D., Mangelsdorf D.J., Shan B. (1999). Identification of a Nuclear Receptor for Bile Acids. Science.

[28] Parks D.J., Blanchard S.G., Bledsoe R.K., Chandra G., Consler T.G., Kliewer S.A., Stimmel J.B., Willson T.M., Zavacki A.M., Moore D.D. (1999). Bile acids: Natural Ligands for an Orphan Nuclear Receptor. Science.

[29] Stofan M., Guo G.L. (2020). Bile Acids and FXR: Novel Targets for Liver Diseases. Front. Med..

[30] Eloranta J.J., Kullak-Ublick G.A. (2008). The Role of FXR In Disorders of Bile Acid Homeostasis. Physiology.

[31] Martinot E., Sédes L., Baptissart M., Holota H., Rouaisnel B., Damon-Soubeyrand C., De Haze A., Saru J., Thibault-Carpentier C., Keime C. (2017). The Bile Acid Nuclear Receptor Fxrα Is a Critical Regulator of Mouse Germ Cell Fate. Stem Cell Rep..

[32] Jung D., Mangelsdorf D.J., Meyer U.A. (2006). Pregnane X Receptor Is a Target of the Farnesoid XD Receptor. J. Biol. Chem..

[33] Modica S., Bellafante E., Moschetta A. (2009). Master Regulation of Bile Acid and Xenobiotic Metabolism Via the FXR, PXR and CAR Trio. Front. Biosci..

[34] Gray M.A., Pollock C.B., Shook L.B., Squires E.J. (2010). Characterization of Porcine Pregnane X Receptor, Farnesoid X Receptor and Their Splice Variants. Exp. Biol. Med..

[35] Pollock C.B., Rogatcheva M.B., Schook L.B. (2007). Comparative Genomics of Xenobiotic Metabolism: A Porcine-Human PXR Gene Comparison. Mamm. Genome.

[36] Gray M.A., Peacock J.N., Squires E.J. (2009). Characterization of the Porcine Constitutive Androstane Receptor (CAR) and Its Splice Variants. Xenobiotica.

[37] Gray M.A., Squires E.J. (2013). Effects of Nuclear Receptor Transactivation on Steroid Hormone Synthesis and Gene Expression in Porcine Leydig Cells. J. Steroid Biochem. Mol. Biol..

[38] Gray M.A., Squires E.J. (2013). Effects of Nuclear Receptor Transactivation on Boar Taint Metabolism and Gene Expression in Porcine Hepatocytes. J. Steroid Biochem. Mol. Biol..

[39] Gunn M.G., Allen P., Bonneau M., Byrne D.V., Cinotti S., Fredriksen B., Hansen L.L., Karlsson A.H., Linder M.G., Lundström K. (2004). Welfare Aspects of the Castration of Piglets. Scientific Report on the Scientific Panel for Animal Health and Welfare on a Request from the Commission Related to Welfare Aspects of the Castration of Piglets. EFSA J..

[40] Gower D.B. (1972). 16-Unsaturated C19 Steroids A Review of Their Chemistry, Biochemistry and Possible Physiological Role. J. Steroid Biochem..

[41] Meadus W.J., Mason J.I., Squires E.J. (1993). Cytochrome P450c17 from the Porcine and Bovine Adrenal Catalyses the Formation of 5,16-Androstadien-3β-Ol from Pregnenolone in the Presence of Cytochrome b_5_. J. Steroid. Biochem. Molec. Biol..

[42] Nakajin S., Takahashi M., Shinoda M., Hall P.F. (1985). Cytochrome B5 Promotes the Synthesis 16-C19 Steroids by Homogenous Cytochrome P-450 C21 Side-Chain Cleavage from Pig Testis. Biochem. Biophys. Res. Commun..

[43] Billen M.J., Squires E.J. (2009). The Role of Porcine Cytochrome B5a And Cytochrome B5b In the Regulation of Cytochrome P45017A1 activities. J. Steroid. Biochem. Molec. Biol..

[44] Lewis D.F. (2004). 57 Varieties: The Human Cytochromes P450. Pharmacogenomics.

[45] Wiercinska P., Lou Y., Squires E.J. (2012). The Roles of Different Porcine Cytochrome P450 Enzymes and Cytochrome B5a In Skatole Metabolism. Animal.

[46] Schelstraete W., De Clerck L., Govaert L., Millercam J., Devreese M., Deforce D., Van Bocxlaer J., Croubels S. (2019). Characterization of Porcine Hepatic and Intestinal Drug Metabolizing CYP450: Comparison with Human Orthologues from A Quantitative, Activity and Selectivity Perspective. Nature.

[47] Babol J., Squires E.J., Lundström K. (1998). Relationship Between Oxidation and Conjugation Metabolism of Skatole in Pig Liver and Concentrations of Skatole in Fat. J. Anim. Sci..

[48] Diaz G.J., Squires E.J. (2000). Metabolism of 3-Methylindole by Porcine Microsomes: Responsible Cytochrome P450 Enzymes. Toxicol. Sci..

[49] Terner M.A., Gilmore W.J., Lou Y., Squires E.J. (2006). The Role of CYP2A And CYP2E1 in the Metabolism of 3-Methylindole in Primary Cultured Porcine Hepatocytes. Drug Metab. Dispos..

[50] Diaz G.J., Squires E.J. (2003). Phase II In Vitro Metabolism of 3-Methylindole Metabolites in Porcine Liver. Xenobiotica.

[51] Brunius C., Vidanarachchi J.K., Tomankova J., Lundström K., Andersson K., Zamaratskaia G. (2016). Skatole Metabolites in Urine as a Biological Marker of Pigs with Enhanced Hepatic Metabolism. Animal.

[52] Diaz J.G., Skordos K.W., Yost G.S., Squires E.J. (1999). Identification of Phase I metabolites of 3-methylindole produced by pig liver microsomes. Drug Metab. Dispos..

[53] Bæk C., Hansen-Møller J., Friis C., Cornett C., Hansen S.H. (1997). Identification of Selected Metabolites of Skatole in Plasma and Urine from Pigs. J. Agric. Food Chem..

[54] Achour B., Barber J., Rostami-Hodjegan A. (2011). Cytochrome P450 Pig Liver Pie: Determination of Individual Cytochrome P450 Isoform Contents in Microsomes from Two Pig Livers Using Liquid Chromatography in Conjunction with Mass Spectrometry. Drug Metab. Dispos..

[55] Moe M., Lien S., Bendixen C., Hedegaard J., Hornshøj H., Berget I., Meuwissen T.H.E., Grindflek E. (2008). Gene Expression Profiles in Liver of Pigs with Extreme High and Low Levels of Androstenone. BMC Vet. Res..

[56] Doran E., Whittington F.W., Wood J.D., McGivan J.D. (2002). Cytochrome P450IIE1 (CYP2E1) is Induced by Skatole, and This Induction is bBocked by Androstenone in Isolated Pig Hepatocytes. Chem. Biol. Interact..

[57] Zamaratskaia G., Gilmore W.J., Lundström K., Squires E.J. (2007). Effect of Testicular Steroids on Catalytic Activities of Cytochrome P450 Enzymes in Porcine Liver Microsomes. Food Chem. Toxicol..

[58] Puccinelli E., Gervasi P.G., La Marca M., Beffy P., Longo V. (2010). Expression and Inducibility by Phenobarbital of CYP2C33, CYP2C42, CYP2C49, CYP2B22, And CYP3As in Porcine Liver, Kidney, Small Intestine, and Nasal Tissues. Xenobiotica.

[59] Laderoute H., Bone C., Squires E.J. (2018). The Sulfoconjugation of Androstenone and Dehydroepiandrosterone by Human and Porcine Sulfotransferase Enzymes. Steroids.

[60] Sinclair P.A., Gilmore W.J., Lin Z., Lou Y., Squires E.J. (2006). Molecular Cloning and Regulation of Porcine *SULT2A1*: Relationship Between SULT2A1 Expression and Sulfoconjugation of Androstenone. J. Mol. Endocrinol..

[61] Bone C., Squires E.J. (2021). The Uptake and Deconjugation of Androstenone Sulfate in the Adipose Tissue of the Boar. Animals.

[62] Schuler G., Dezhkam Y., Tenbusch L., Klymiuk M.C., Zimmer B., Hoffmann B. (2018). Formation and Hydrolysis of Sulfonated Estrogens in the Porcine Testis and Epididymis. J. Mol. Endocrinol..

[63] Lee F.Y., de Aguiar Vallim T.Q., Chong H.K., Zhang Y., Liu Y., Jones S.A., Osbourne T.F., Edwards P.A. (2010). Activation of the Farnesoid X Receptor Provides Protection Against Acetaminophen-Induced Hepatic Toxicity. Mol. Endocrinol..

[64] Dasgupta S., Lonard D.M., O’Malley B.W. (2014). Nuclear Receptor Coactivators: Master Regulators of Human Health and Disease. Annu. Rev. Med..

[65] Sun Z., Xu Y. (2020). Nuclear Receptor Coactivators (NCOAs) and Corepressors (NCORs) in the Brain. Endocrinology.

[66] Heery D.M., Kalkhoven E., Hoare S., Parker M.G. (1997). A Signature Motif in Transcriptional Co-Activators Mediates Binding to Nuclear Receptors. Nature.

[67] Le Douarin B., Nielsen L.A., Garnier J., Ichinose H., Jeanmougin F., Losson R., Chambon P.A. (1996). Possible Involvement of TIF1α and TIF1β In the Epigenetic Control of Transcription by Nuclear Receptors. EMBO J..

[68] Dasgupta S., O’Malley B.W. (2014). Transcriptional Coregulators: Emerging Roles of SRC Family of Coactivators in Disease Pathology. J. Mol. Endocrinol..

[69] Perissi V., Rosenfeld M.G. (2005). Controlling Nuclear Receptors: The Circular Logic of Cofactor Cycles. Nat. Rev. Mol. Cell Biol..

[70] Rosenfeld M.G., Lunyak V.V., Glass C.K. (2006). Sensors and Signals: A Coactivator/Corepressor/Epigenetic Code for Integrating Signal-Dependent Programs of Transcriptional Response. Genes Dev..

[71] Pavek P. (2016). Pregnane X receptor (PXR)–Mediated Gene Repression and Cross-Talk of PXR with Other Nuclear Receptors Via Coactivator Interactions. Front. Pharmacol..

[72] Hu X., Lazar M.A. (1999). The Cornr Motif Controls the Recruitment of Corepressors by Nuclear Hormone Receptors. Nature.

[73] Nagy L., Kao H., Love J.D., Li C., Banayo E., Cooch J.T., Krishna V., Chatterjee K., Evans R.M., Schwabe W.R. (1999). Mechanism of Corepressor Binding and Release from Nuclear Hormone Receptors. Genes Dev..

[74] Goodson M., Jonas B.A., Privalsky M.A. (2005). Corepressors: Custom Tailoring and Alterations While You Wait. Nucl. Recept. Signal..

[75] Baptissart M., Martinot E., Vega A., Sédes L., Rouaisnel B., de Haze A., Baron S., Schoonjas K., Caira F., Volle D.H. (2016). Bile Acid-Fxrα Pathways Regulate Male Sexual Maturation in Mice. Oncotarget.

[76] Ourlin J.C., Lasserre F., Pineau T., Fabre J.M., Sa-Cunha A., Maurel P., Vilarem M.J., Pascussi J.M. (2003). The Small Heterodimer Partner Interacts with the Pregnane X Receptor and Represses Its Transcriptional Activity. Mol. Endocrinol..

[77] Li T., Chiang J.Y.L. (2006). Rifampicin Induction of CYP3A4 Requires Pregnane X Receptor Cross Talk with Hepatocyte Nuclear Factor 4α and Coactivators, and Suppression of Small Heterodimer Partner Gene Expression. Drug Metab. Dispos..

[78] Saini S.P.S., Mu Y., Gong H., Toma D., Uppal H., Ren S., Li S., Poloyac S.M., Xie W. (2005). Dual Role of Orphan Nuclear Receptor Pregnane X Receptor in Bilirubin Detoxification in Mice. Hepatology.

[79] Pascussi J.M., Gerbal-Chaloin S., Duret C., Daujat-Chavanieu M., Vilarem M.J., Maurel P. (2008). The Tangle of Nuclear Receptors That Controls Xenobiotic Metabolism and Transport: Crosstalk and Consequences. Annu. Rev. Pharmacol. Toxicol..

[80] Masuyama H., Hiramatsu Y., Kunitomi M., Kudo T., MacDonald P.N. (2000). Endocrine Disrupting Chemicals, Phthalic Acid and Nonylphenol, Activate Pregnane X Receptor-Mediated Transcription. Mol. Endocrinol..

[81] Quigley E.M.M. (2013). Gut Bacteria in Health and Disease. Gastroenterol. Hepatol..

[82] Dempsey J.L., Cui J.Y. (2019). Microbiome is a Functional Modifier of P450 Drug Metabolism. Curr. Pharmacol. Rep..

[83] Jimenez A.G., Sperandio V., Tommonaro G. (2019). Quorum Sensing and the Gut Microbiome. Quorum Sensing.

[84] Li X., Zhang B., Hu Y., Zhao Y. (2021). New Insights into Gut-Bacteria-Derived Indole and Its Derivatives in Intestinal and Liver Diseases. Front. Pharmacol..

[85] Albillos A., de Gottardi A., Rescigno M. (2020). The Gut-Liver Axis in Liver Disease: Pathophysiological Basis for Therapy. J. Hepatol..

[86] Nielsen S.D., Bauhaus Y., Zamaratskaia G., Junqueira M.A., Blaabjerg K., Petrat-Melin B., Young J.F., Rasmussen M.K. (2017). Constitutive Expression and Activity of Cytochrome P450 In Conventional Pigs. Res. Vet. Sci..

[87] Dodd D., Spitzer M.H., Van Treuren W., Merrill B.D., Hryckowian A.J., Higginbottom S.K., Le A., Cowan T.M., Nolan G.P., Fischbach M.A. (2017). A Gut Bacterial Pathway Metabolizes Aromatic Amino Acids into Nine Circulating Metabolites. Nature.

[88] Elsden S.R., Hilton M.G., Waller J.M. (1976). The End Products of the Metabolism of Aromatic Amino Acids by Clostridia. Arch. Microbiol..

[89] Wlodarska M., Luo C., Kolde R., d’Hennezel E., Annand J.W., Heim C.E., Krastel P., Schmitt E.K., Omar A.S., Creasey E. (2017). Indoleacrylic Acid Produced by Commensal *Peptostreptococcus* Species Suppresses Inflammation. Cell Host Microbe.

[90] Rasmussen M.K., Daujat-Chavanieu M., Gerbal-Chaloin S. (2017). Activation of the Aryl Hydrocarbon Receptor Decreases-Rifampicin-Induced CYP3A4 Expression in Primary Human Hepatocytes and Heparg. Toxicol. Lett..

[91] Sári Z., Mikó E., Kovács T., Jankó L., Csonka T., Lente G., Sebő É., Tóth J., Tóth D., Árkosy P. (2020). Indolepropionic Acid, A Metabolite of the Microbiome, Has Cytostatic Properties in Breast Cancer by Activating AHR and PXR Receptors and Inducing Oxidative Stress. Cancers.

[92] Morgan E.T., Dempsey J.L., Mimche S.M., Lamb T.J., Kulkarni S., Cui J.Y., Jeong H., Slitt A.L. (2018). Physiological Regulation of Drug Metabolism and Transport: Pregnancy, Microbiome, Inflammation, Infection, and Fasting. Drug Metab. Depos..

[93] Illés P., Krasulová K., Vyhlídalová B., Poulíková K., Marcalíková A., Pečinková P., Sirotová N., Vrzal R., Mani S., Dvořák Z. (2020). Indole Microbial Intestinal Metabolites Expand the Repertoire of Ligands and Agonists of The Human Pregnane X Receptor. Toxicol. Lett..

[94] Lee J.H., Lee J. (2010). Indole as an Intercellular Signal in Microbial Communities. FEMS Microbiol. Rev..

[95] Tsavkelova E., Oeser B., Oren-Young L., Israeli M., Sasson Y., Tudzynski B., Sharon A. (2012). Identification and Functional Characterization of Indole-3-Acetamide-Mediated IAA Biosynthesis in Plant-Associated *Fusarium* species. Fungal Genet. Biol..

[96] Jensen B.B. (2006). Prevention of Boar Taint in Pig Production. Factors Affecting the Level of Skatole. Acta Vet. Scand..

[97] Zelante T., Iannitti R.G., Cunha C., De Luca A., Giovannini G., Pieraccini G., Zecchi R., D’Angelo C., Massi-Benedetti C., Fallarino F. (2013). Tryptophan Catabolites from Microbiota Engage Aryl Hydrocarbon Receptor and Balance Mucosal Reactivity Via Interlukin-22. Immunity.

[98] Whitehead T.R., Price N.P., Drake H.L., Cotta M.A. (2008). Catabolic Pathway for the Production of Skatole and Indoleacetic Acid by the Acetogen *Clostridium drakei*, *Clostridium scatologenes*, and Swine Manure. Appl. Environ. Microbiol..

[99] Li X., Jensen R.L., Højberg O., Canibe N., Jensen B.B. (2015). *Olsenella scatoligenes* sp. Nov., a 3-methylindole- (skatole) and 4-methylphenol-(*p*-cresol) Producing Bacterium Isolated from Pig Faeces. Int. J. Syst. Evol. Microbiol..

[100] Vyhlídalová B., Krasulová K., Pečinková P., Marcalíková A., Vrzal R., Zemánková L., Vančo J., Trávníček Z., Vondráček J., Karasová M. (2020). Gut Microbial Catabolites of Tryptophan Are Ligands and Agonists of the Aryl Hydrocarbon Receptor: A Detailed Characterization. Int. J. Mol. Sci..

[101] Rasmussen M.K., Klausen C.L., Ekstrand B. (2014). Regulation of Cytochrome P450 Mrna Expression in Primary Porcine Hepatocytes by Selected Secondary Plant Metabolites from Chicory (*Cichorium intybus* L.). Food Chem..

[102] Monshouwer M., van’t Klooster G.A.E., Nijmeijer S.M., Witkamp R.F., van Miert A.S.J.P.A.M. (1998). Characterization of Cytochrome P450 Isoenzymes in Primary Cultures of Pig Hepatocytes. Toxicol. Vitr..

[103] Richards L.B., Li M., van Esch B.C.A.M., Garssen J., Folkerts G. (2016). The Effects of Short-Chain Fatty Acids on the Cardiovascular System. PharmaNutrition.

[104] Venegas D.P., De la Fuente M., Landskron G., González M.J., Quera R., Dijkstra G., Harmsen J.M., Faber K.N., Hermoso M.A. (2019). Short Chain Fatty Acids (Scfas)-Mediated Gut Epithelial and Immune Regulation and Its Relevance for Inflammatory Bowel Diseases. Front. Immunol..

[105] den Besten G., Bleeker A., Gerding A., van Eunen K., Havinga R., van Dijk T.H., Oosterveer M.H., Jonker J.W., Groen A.K., Reijngoud D.J. (2015). Short-Chain Fatty Acids Protect Against High-Fat Diet-Induced Obesity Via Pparγ-Dependent Switch from Lipogenesis to Fat Oxidation. Diabetes.

[106] Jung T.H., Park J.H., Jeon W.M., Han K.S. (2015). Butyrate Modulates Bacterial Adherence on LS174T Human Colorectal Cells by Stimulating Mucin Secretion and MAPK Signaling Pathway. Nutr. Res. Pract..

[107] Tan J., Mckenzie C., Potamitis M., Thorburn A.N., Mackay C.R., Macia L., Alt F., Murphy K. (2014). The Role of Short-Chain Fatty Acids in Health and Disease. Advances in Immunology.

[108] Jansen M.S., Nagel S.C., Miranda P.J., McDonnell D.P. (2004). Short-Chain Fatty Acids Enhance Nuclear Receptor Activity Through Mitogen-Activated Protein Kinase Activation and Histone Deacetylase Inhibition. Proc. Natl. Acad. Sci. USA..

[109] Jin U.H., Park H., Davidson L.A., Callaway E.S., Chapkin R.S., Jayaraman A., Asante A., Allred C., Weaver E.A., Safe S. (2017). Short Chain Fatty Acids Enhance Aryl Hydrocarbon (Ah) Responsiveness in Mouse Colonocytes and Caco-2 Human Colon Cancer Cells. Nature.

[110] Marinelli L., Martin-Gallausiaux C., Bourhis J.M., Béguet-Crespel F., Blottière H.M., Lapaque N. (2019). Identification of the Novel Role of Butyrate as Ahr Ligand in Human Intestinal Epithelial Cells. Nature.

[111] Ranhotra H.S., Flannigan K.L., Brave M., Mukherjee S., Lukin D.J., Hirota S.A., Mani S. (2016). Xenobiotic Receptor-Mediated Regulation of Intestinal Barrier Function and Innate Immunity. Nucl. Receptor Res..

[112] Ren J., Sun K., Wu Z., Yao J., Guo B. (2011). All 4 Bile Salt Hydrolase Proteins Are Responsible for the Hydrolysis Activity in *Lactobacillus plantarum* ST-III. J. Food Sci..

[113] Elkins C.A., Moser S.A., Savage D.C. (2001). Genes Encoding Bile Salt Hydrolases and Conjugated Bile Salt Transporters in *Lactobacillus johnsonii* 100-100 and other *Lactobacillus* species. Microbiology.

[114] Jayashree S., Pooja S., Pushpanathan M., Rajendhran J., Gunasekaran P. (2014). Identification and Characterization of Bile Salt Hydrolase Genes from the Genome of *Lactobacillus fermentum* MTCC 8711. Appl. Biochem. Biotechnol..

[115] Franz C.M.A.P., Specht I., Haberer P., Holzapfel W.H. (2001). Bile Salt Hydrolase Activity of Enterococci Isolated from Food: Screening and Quantitative Determination. J. Food Prot..

[116] Tanaka H., Hashiba H., Kok J., Mierau I. (2000). Bile Salt Hydrolase of *Bifidobacterium Longum*–Biochemical and Genetic Characterization. Appl. Environ. Microbiol..

[117] Kim G.B., Yi S.H., Lee B.H. (2004). Purification and Characterization of Three Different Types of Bile Salt Hydrolases from Bifidobacterium Strains. J. Dairy Sci..

[118] Rossocha M., Schultz-Heienbrok R., von Moeller H., Coleman J.P., Saenger W. (2005). Conjugated Bile Acid Hydrolase Is a Tetrameric N-Terminal Thiol Hydrolase with Specific Recognition of Its Cholyl but Not of Its Tauryl Product. Biochemistry.

[119] Jones B.V., Begley M., Hill C., Gahan C.G.M., Marchesi J.R. (2008). Functional and Comparative Metagenomics Analysis of Bile Salt Hydrolase Activity in the Human Gut Microbiome. Proc. Natl. Acad. Sci. USA.

[120] Reichen J., Paumgartner G. (1976). Uptake of Bile Acids by Perfused Rat Liver. Am. J. Physiol..

[121] Salvioli G., Lugli R., Pradelli J.M., Gigliotti G. (1985). Bile Acid Binding in Plasma: The Importance of Lipoproteins. FEBS Lett..

[122] Grüner N., Mattner J. (2021). Bile Acids and Microbiota: Multifaceted and Versatile Regulators of the Liver-Gut Axis. Int. J. Mol. Sci..

[123] Ajouz H., Mukherji D., Shamseddine A. (2014). Secondary Bile Acids: An Underrecognized Cause of Colon Cancer. J. Surg. Oncol..

[124] Urdaneta V., Cassadesús J. (2017). Interactions Between Bacteria and Bile Salts in the Gastrointestinal and Hepatobiliary Tracts. Front. Med..

[125] Jiang L., Zhang H., Xiao D., Wei H., Chen Y. (2021). Farnesoid X Receptor (FXR): Structures and Ligands. Comput. Struct. Biotechnol. J..

[126] Staudinger J.L., Goodwin B., Jones S.A., Hawkins-Brown D., MacKenzie K.I., LaTour A., Liu Y., Klaassen C.D., Brown K.K., Reinhard J. (2001). The Nuclear Receptor PXR Is a Lithocholic Acid Sensor That Protects Against Liver Toxicity. Proc. Natl. Acad. Sci. USA.

[127] Barnes S., Buchina E.S., King R.J., McBurnett T., Taylor K.B. (1989). Bile Acid Sulfotransferase I From Rat Liver Sulfates Bile Acids And 3-Hydroxy Steroids: Purification, N-Terminal Amino Acid Sequence, and Kinetic Properties. J. Lipid Res..

[128] Pillot T., Ouzzine M., Fournel-Gigleux S., Lafaurie C., Radominska A., Burchell B., Siest G., Magdalou J. (1993). Glucuronidation of Hyodeoxycholic Acid in Human Liver. J. Biol. Chem..

[129] Claudel T., Staels B., Kuipers F. (2005). The farnesoid X Receptor a Molecular Link Between Bile Acid and Lipid and Glucose Metabolism. Arterioscler. Thromb. Vasc. Biol..

[130] Moore L.B., Maglich J.M., McKee D.D., Wisely B., Wilson T.M., Kliewer S.A., Lambert M.H., Moore J.T. (2002). Pregnane X Receptor (PXR), Constitutive Androstane Receptor (CAR), And Benzoate X Receptor (BXR) Define Three Pharmacologically Distinct Classes of Nuclear Receptors. Mol. Endocrinol..

[131] Jen K., Squires E.J. (2011). Efficacy of Non-Nutritive Sorbent Materials as Intestinal-Binding Agents for the Control of Boar Taint. Animal.

[132] Claus R., Lösel D., Lacorn M., Mentshel J., Schenkel H. (2003). Effects of Butyrate on Apoptosis in the Pig Colon and Its Consequences for Skatole Formation and Tissue Accumulation. J. Anim. Sci..

[133] Li X., Jensen B.B., Canibe N. (2019). The Mode of Action of Chicory Roots on Skatole Production in Entire Male Pigs Is Neither Via Reducing the Population of Skatole-Producing Bacteria nor Via Increased Butyrate Production in the Hindgut. Appl. Environ. Microbiol..

[134] Singh B., Halestrap A.P., Paraskeva C. (1997). Butyrate Can Act as a Stimulator of Growth or Inducer of Apoptosis in Human Colonic Epithelial Cell Lines Depending on the Presence of Alternative Energy Sources. Carcinogenesis.

[135] Knarreborg A., Beck J., Jensen M.T., Laue A., Agergaard N., Jensen B.B. (2002). Effect of Non-Starch Polysaccharides on Production and Absorption of Indolic Compounds in Entire Male Pigs. Anim. Sci..

[136] Whittington F.M., Nute G.R., Hughes S.I., McGivan J.D., Lean I.J., Wood J.D., Doran E. (2004). Relationship Between Skatole and Androstenone Accumulation, And Cytochrome P4502E1 Expression in Meishan X Large White Pigs. Meat Sci..

[137] Rasmussen M.K., Zamaratskaia G., Ekstrand B. (2011). In Vivo Effect of Dried Chicory Root (*Cichorium Intybus* L.) on Xenobiotica Metabolizing Cytochrome P450 Enzymes in Porcine Liver. Toxicol. Lett..

[138] Simonsson U.S.H., Lindell M., Raffalli-Mathieu F., Lannerbro A., Honkakoski P., Lang M.A. (2006). In Vivo and Mechanistic Evidence of Nuclear Receptor CAR Induction by Artemisinin. Eur. J. Clin. Investig..

[139] Sachar M., Ma X. (2013). Nuclear Receptors in Herb-Drug Interactions. Drug Metab. Rev..

[140] Hernandez J.P., Mota L.C., Baldwin W.S. (2009). Activation of CAR and PXR by dietary, Environmental and Occupational Chemicals Alters Drug Metabolism, Intermediary Metabolism and Cell Proliferation. Curr. Pharmacogenomics Person. Med..

[141] Liu W., Wong C. (2010). Oleanolic Acid Is a Selective Farnesoid X Receptor Modulator. Phytother. Res..

[142] Le Bon A.M., Vernevaut M.F., Guenot L., Kahane R., Auger J., Arnault I., Haffner T., Siess M.H. (2003). Effects of Garlic Powders with Varying Alliin Contents on Hepatic Drug Metabolizing Enzymes in Rats. J. Agric. Food Chem..

[143] Cherrington N.J., Slitt A.L., Maher J.M., Zhang X.X., Zhang J., Huang W., Wan Y.J.Y., Moore D.D., Klaassen C.D. (2003). Induction of Multidrug Resistance Protein 3 (MRP3) *In Vivo* Is Independent of Constitutive Androstane Receptor. Drug Metab. Dispos..

[144] Moore L.B., Goodwin B., Jones S.A., Wisely G.B., Serabjit-Singh C.J., Willson T.M., Collins J.L., Kliewer S.A. (2000). St. John’s Wort Induces Hepatic Drug Metabolism Through Activation of the Pregnane X Receptor. Proc. Natl. Acad. Sci. USA.

[145] Wang H., Li H., Moore L.B., Johnson M.D.L., Maglich J.M., Goodwin B., Ittoop O.R.R., Wisely B., Creech K., Parks D.J. (2008). The Phytoestrogen Coumestrol Is a Naturally Occurring Antagonist of the Human Pregnane X Receptor. Mol. Endocrinol..

[146] Rajaraman G., Chen J., Chang T.K.H. (2006). Ginkgolide A Contributes to the Potentiation of Acetaminophen Toxicity by *Ginkgo Biloba* Extract in Primary Cultures of Rat Hepatocytes. Toxicol. Appl. Pharmacol..

[147] Chang T.K.H., Chen J., Teng X.W. (2006). Distinct Role of Bilobalide and Ginkgolide in the Modulation of Rat CYP2B1 and CYP3A23 Gene Expression by *Ginkgo biloba* Extract in Cultured Hepatocytes. Drug Metab. Dispos..

[148] Li L., Stanton J.D., Tolson A.H., Luo Y., Wang H. (2009). Bioactive Terpenoids and Flavonoids from Ginkgo Biloba Extract Induce the Expression of Hepatic Drug-Metabolizing Enzymes Through Pregnane X Receptor, Constitutive Androstane Receptor, and Aryl Hydrocarbon Receptor-Mediated Pathways. Pharm. Res..

[149] Brobst D.E., Ding X., Creech K.L., Goodwin B., Kelley B., Staudinger J.L. (2004). Guggulsterone Activates Multiple Nuclear Receptors and Induces CYP3A Gene Expression Through the Pregnane X Receptor. J. Pharmacol. Exp. Ther..

[150] Ding X., Staudinger J.L. (2005). The Ratio of Constitutive Androstane Receptor to Pregnane X Receptor Determines the Activity of Guggulsterone Against the Cyp2b10 Promoter. J. Pharmacol. Exp. Ther..

[151] Feng W., Ao H., Peng C. (2018). Gut Microbiota, Short-Chain Fatty Acids, And Herbal Medicines. Front. Pharmacol..

[152] Bachs L., Parés A., Elena M., Piera C., Rodsés J. (1992). Effects of Long-Term Rifampicin Administration in Primary Biliary Cirrhosis. Gastroenterology.

[153] Kurian R., Hedrich W., Mackowiak B., Li L., Wang H. (2020). CITCO as an Adjuvant Facilitates CHOP-Based Lymphoma Treatment in Hcar-Transgenic Mice. Cells.

[154] Song M., Zhang F., Chen L., Yang Q., Su H., Yang X., He H., Ling M., Zheng J., Duan C. (2021). Dietary Chenodeoxycholic Acid Improves Growth Performance and Intestinal Health by Altering Serum Metabolic Profiles and Gut Bacteria in Weaned Piglets. Anim. Nutr..

[155] Burkina V., Rasmussen M.K., Oliinychenko Y., Zamaratskaia G. (2019). Porcine Cytochrome 2A19 and 2E1. Basic Clin. Pharmacol. Toxicol..

[156] Squires E.J., Bone C., Cameron J. (2020). Pork Production with Entire Males: Directions for Control of Boar Taint. Animals.

[157] Drag M.H., Kogelman L.J.A., Maribo H., Meinert L., Thomsen P.D., Kadarmideen H.N. (2019). Characterization of Eqttls Associated with Androstenone by RNA Sequencing in Porcine Testis. Physiol. Genom..

[158] Drag M., Hansen M.B., Kadarmideen H.N. (2018). Systems Genomics Study Reveals Expression Quantitative Trait Loci, Regulator Genes and Pathways Associated with Boar Taint in Pigs. PLoS ONE.

[159] Drag M., Skinkyté-Juskiené R., Do D.N., Kogelman L.J.A., Kadarmideen H.N. (2017). Differential Expression and Co-Expression Gene Networks Reveal Candidate Biomarkers of Boar Taint in Non-Castrated Pigs. Sci. Rep..

[160] Duarte D.A., Schroyen M., Mota R.R., Vanderick S., Gengler N. (2021). Recent Advances on Boar Taint Reduction as an Alternative to Castration: A Review. J. Appl. Genet..

